# Assessment of serum granulysin and cathepsin-L levels in vitiligo patients

**DOI:** 10.1590/1806-9282.20231107

**Published:** 2024-05-20

**Authors:** Cemre Yazar, Atiye Akbayrak, Zeliha Cansel Ozmen, Yunus Emre Kuyucu, Mehtap Sencan, Omer Kutlu, Havva Yildiz Seckin

**Affiliations:** 1 Gaziosmanpasa University, School of Medicine, Department of Dermatology and Venereology – Tokat, Turkey.; 2 Gaziosmanpasa University, School of Medicine, Department of Biochemistry – Tokat, Turkey.; 3 Gaziosmanpasa University, School of Medicine, Department of Biostatistics – Tokat, Turkey.; 4 Dortyol State Hospital – Dörtyol, Turkey.

**Keywords:** Vitiligo, Granulysin, Cathepsin L, Autoimmunity, Cellular immunity

## Abstract

**OBJECTIVE:**

Cellular and humoral immunity plays a role in the pathogenesis of vitiligo. T lymphocytes and natural killer cells involved in cellular immunity carry out their cytotoxic activities through perforin/granzyme-dependent granule exocytosis, in which granulysin and cathepsin-L are also involved. The aim of this study was to investigate the possible role of serum granulysin and cathepsin-L in the etiopathogenesis of vitiligo and their association with disease activity and severity.

**METHODS:**

This randomized, prospective case-control study was conducted with 46 vitiligo patients admitted to the hospital for vitiligo between January and November 2021 and 46 healthy volunteers of similar age and gender. Serum levels of granulysin and cathepsin-L were measured by the enzyme-linked immunosorbent assay method.

**RESULTS:**

The mean serum levels of granulysin and cathepsin-L were statistically significantly higher in vitiligo patients compared with the control group (p=0.048 and p=0.024, respectively). There was no statistically significant correlation between serum granulysin and serum cathepsin-L levels and disease severity in the patient group (r=0.30, p=0.062 and r=0.268, p=0.071, respectively). Disease activity also showed no significant association with serum granulysin and cathepsin-L levels (p=0.986 and p=0.962, respectively).

**CONCLUSION:**

Although granulysin and cathepsin-L are molecules involved in the pathogenesis of vitiligo, the use of these molecules may not be helpful in assessing disease activity and severity. It may be helpful to conduct comprehensive and prospective studies to find new molecules to fill the gap in this area.

## INTRODUCTION

Vitiligo is an acquired systemic disease characterized by selective destruction of melanocytes^1,2^. Although etiopathogenesis is not clearly known, many theories (genetics, autoimmunity, oxidative stress, production of inflammatory mediators, and melanocyte separation mechanisms) have been proposed to explain the pathogenesis^3^. Among these theories, the autoimmunity theory is the most widely accepted^4^.

Both innate immunity and acquired immunity play a role in the pathogenesis of vitiligo^5^. Specifically, natural killer (NK) cells play the main role in innate immunity and melanocyte-specific CD8+ cytotoxic T lymphocytes (CTLs) in acquired immunity^5,6^. Both cells exert their cytotoxic activities through the cytolytic molecules they contain in large amounts^7^.

Granulysin is a cytolytic protein found in the granules of CTLs and NK cells, acting synergistically with perforin and inducing apoptosis^7,8^. Serum granulysin level is considered a useful indicator that can be used to assess cytotoxic immunity^7^.

Cathepsin-L, which is a member of the lysosomal cysteine protease family, plays an important role in regulating the immune response by contributing to antigen presentation, adhesion, and migration, as well as cytokine and growth factor degradation^9,10^. It is suggested that this molecule may be a useful indicator for assessing cellular immunity^11^.

This study investigates whether granulysin and cathepsin-l could be indicators of cell-mediated cytotoxicity in the pathogenesis of vitiligo and whether their levels measured in the acute phase could be markers that can help predict disease activity and prognosis of vitiligo.

## METHODS

This randomized prospective case-control study was conducted in a single center between January and November 2021. A total of 46 vitiligo patients and 46 healthy volunteers of similar age and gender were included in the study. Before the study, ethical approval (20-CREC-297) was obtained from the clinical research ethics committee. All patients and healthy volunteers included in the study gave their verbal and written informed consent. The rules of the Declaration of Helsinki of the World Medical Association were followed during the study. Subjects between the ages of 18 and 65 years diagnosed with vitiligo were included in the study. Subjects with findings/diseases that could affect serum levels of granulysin and cathepsin-L (allergic, autoimmune, and/or chronic inflammatory systemic and/or skin diseases, phototherapy in the past 3 months, infections in the past 1 month, immunosuppression, or malignancy) and pregnant and lactating subjects were excluded from the study.

A dermatologic examination of all study participants was performed by the same physician. In addition to demographic characteristics such as age, gender, and body mass index (BMI) of the patient and control groups, the presence of autoimmune diseases (including vitiligo) and atopy in the family was asked, and the data were recorded. In addition, in the patient group, vitiligo onset age, localization, disease duration, vitiligo subtype (diffuse, acrofacial, or focal), koebnerization, mucosal involvement, leukotrichia, and presence of halo nevus were determined by interview.

The vitiligo area severity index (VASI) was used to evaluate the disease severity of the patient group, and the vitiligo disease activity score (VIDA) was used to evaluate disease activity. If vitiligo started before 10 years of age, it was defined as early onset, and if it started after 10 years of age, it was defined as late onset^12^. Those with a VIDA score of <0 were classified as stable, and those with a VIDA score of >1 were classified as active vitiligo^13^.

The patient and control groups underwent clinical and laboratory evaluation (e.g., fasting blood glucose, TSH, free T4, antithyroglobulin antibody, antithyroid peroxidase antibody, vitamin B12, and hemogram) to rule out autoimmune, allergic, and/or chronic inflammatory systemic and/or skin diseases.

### Measurement of granulysin and cathepsin-L in serum

Peripheral venous blood samples placed in a gel biochemistry tube after fasting for at least 8 h were stored at room temperature for 20 min to coagulate and then centrifuged at 3,000 rpm for 10 min. After centrifugation, serum was separated, filled into Eppendorf tubes, and stored at −80°C until the day of the study.

Serum samples brought to room temperature before the study day were analyzed separately using the enzyme-linked immunosorbent assay (ELISA) method.

For the measurement of granulysin in serum, the BT LAB brand ELISA kit for human granulysin was used with a reference range of 0.5–100 ng/mL; for the measurement of cathepsin-L in serum, the BT LAB brand ELISA kit for human cathepsin-L was used with a reference range of 0.1–40 ng/mL.

### Statistical analysis

Descriptive analyses were performed for the general characteristics of the study groups. Categorical data were presented as numbers (n) and percentages (%). When numerical data were normally distributed, they were expressed as mean±standard deviation, and when they were not normally distributed, they were expressed as median (min–max). The association between continuous variables was determined with the Pearson correlation test; the agreement of variables with normal distribution was assessed with the Kolmogorov-Smirnov test. For the comparison of continuous variables by groups, the independent-sample t-test and the one-way analysis of variance (ANOVA) were used as parametric tests; the Kruskal-Wallis test and the Mann-Whitney U test were used as non-parametric tests. Comparison of categorical variables by groups was performed with the chi-square test and Fisher's exact chi-square test.

All analyses were performed with the Statistical Package for Social Sciences program (SPSS Inc., Chicago, IL) version 20.0. Statistical significance was accepted as p<0.05 in all comparisons.

## RESULTS

A total of 46 vitiligo patients and 46 healthy volunteers were included in the study. There were 24 women (52.2%) and 22 men (47.8) in the patient group and 23 women (50%) and 23 men (50%) in the control group. The mean ages of the patient and control groups were 31.22±10.91 and 32.72±9.47 years, respectively (p=0.483), and the age and gender distributions of the patient and control groups were similar (p=0.835). The family history of vitiligo was significantly higher in the patient group than in the control group (p=0.003).

When vitiligo was classified according to clinical subtypes, diffuse vitiligo was present in 27 patients (58.7%), acral/acrofacial vitiligo in 12 patients (26.1%), and focal vitiligo in 7 patients (15.2%). Three of the patients (6.5%) had early-onset vitiligo, and 43 (93.5%) had late-onset vitiligo. The mean disease duration was 5.51±5.01 years (0.08–19 years), and the disease onset age was 25.84±12.12 years (6–55.75 years).

The clinical characteristics of vitiligo patients are shown in Table 1.

**Table 1:** Clinical characteristics of vitiligo patients.

Characteristic		n (%)
Vitiligo subtype	Diffuse	27 (58.7)
	Acral/acrofacial	12 (26.1)
	Focal	7 (15.2)
Disease onset age	Early onset (<10 years)	3 (6.5)
	Late onset (>10 years)	43 (93.5)
Disease duration	<15 years	43 (93.5)
	>15 years	3 (6.5)
Disease activity status	Active	31 (67.4)
	Stable	15 (32.6)
Halo nevus	(+)	2 (4.3)
	(-)	44 (95.7)
Leukotrichia	(+)	25 (54.3)
	(-)	21 (45.7)
Koebnerization	(+)	10 (21.7)
	(-)	36 (78.3)
Mucosal involvement	(+)	9 (19.6)
	(-)	37 (80.4)

+ present;

- absent.

The median VASI score of the patients was 1 (0.1–46.15), VIDA score was −1 in 10.9%, 0 in 21.7%, +1 in 2.2%, +2 in 17.4%, +3 in 15.2%, and +4 in 32.6%.

Serum levels of granulysin and cathepsin-L were statistically significantly higher in the patient group than in the control group (p=0.048 and p=0.024, respectively) (Table 2).

**Table 2:** Serum granulysin and cathepsin-L levels of the patient and control groups.

	Patient (median±SD)	Control (median±SD)	p
Serum granulysin level (ng/mL)	45.53±31.42	34.26±21.53	**0.048**
Serum cathepsin-L level (ng/mL)	13.98±10.84	9.61±6.92	**0.024**

Statistically significant p-values are denoted in bold.

There was no statistically significant correlation between serum granulysin and cathepsin-L levels and age, BMI, disease onset age, duration, and severity in the patient group.

No statistically significant association was found between serum granulysin and cathepsin-L levels and gender, Fitzpatrick skin type, concomitant halo nevus, leukotrichia, mucosal involvement, presence of Koebner, disease activity, and vitiligo subtype (p>0.05).

A very high positive correlation was found between serum granulysin and cathepsin-L levels in vitiligo patients (Table 3).

**Table 3:** Correlation between serum granulysin and cathepsin-L levels in the patient group.

		Serum cathepsin-L level (ng/mL)
Serum granulysin level (ng/mL)	r	0.975
	p	**0.000**

Statistically significant p-value is denoted in bold.

## DISCUSSION

Vitiligo is a disease whose treatment and clinical follow-up are difficult because its etiopathogenesis is not clear^14^. There is a need for molecules and parameters that will help clinicians overcome these difficulties and provide clues to disease severity, activity status, and clinical course. There are a very limited number of recent studies on the possible role of granulysin and cathepsin-L in the etiopathogenesis of vitiligo and as indicators of disease severity and activity^15,16^. To the best of our knowledge, this study is the first to simultaneously investigate the possible role of these two molecules in the pathogenesis of vitiligo as indicators of cellular immunity and cytotoxicity.

Vitiligo is a chronic, acquired, systemic disease that affects melanocytes in the body, particularly in the basal layer of the epidermis, and takes an unpredictable clinical course^1,2^. Although the pathogenesis is not fully understood, the autoimmune theory is the most widely accepted theory on the subject^17^. The efficacy of biological and targeted therapies used in vitiligo patients in recent years supports the autoimmune theory^18,19^.

Both cellular immunity and humoral immunity play a role in the pathogenesis of vitiligo, an autoimmune disease. Recent studies support the view that specific cytotoxic T cells (CD8+ T cells) are responsible for the destruction of melanocytes by releasing mainly type 1 cytokines such as TNF α and IFN-γ^20,21^. CXC chemokines such as CXCL 9, CXCL10, and CXCL11, which are induced by IFN-γ, are those most strongly associated with the recruitment of CTLs in the epidermis. These chemokines act on melanocytes by binding to the CXCR3 receptor, and in recent years, studies in mouse models have shown the importance of the IFN-γ-CXCL10-CXCR3 axis in melanocyte destruction in vitiligo^22,23^.

Cytotoxicity is an essential component of the cell-mediated immune system and is a multifactorial process. NK cells and CD8+ T cells are the two major cytotoxic cell populations. NK and CD8+ T cells initiate targeted cell death by inducing apoptosis. There are many cytolytic molecules stored in granules that initiate apoptosis, including perforin, granzyme, granulysin, and cathepsin-L^24–26^. The granulysin level in serum is considered an important indicator for the assessment of cytotoxic immunity^7^.

In a recent study, Mohammed et al.^15^ investigated granulysin levels in vitiligo and found that serum granulysin levels are higher in vitiligo patients than in healthy volunteers, similar to this study, and that the molecular level is not associated with disease severity and activity. In another recent study, granulysin levels were found to be higher in the patient group than in healthy volunteers, and unlike this study, a positive correlation was found between the molecule and disease severity and activity^16^.

Oba et al.^27^ from our country, who examined serum granulysin levels before and after treatment with tofacitinib in patients with alopecia areata, found that the serum granulysin levels decreased significantly after treatment compared with baseline and that this molecule level was correlated with the immunological activity of alopecia areata. Similar to alopecia areata patients, the serum granulysin level in this study was also statistically significantly higher in the patient group than in the control group. However, no correlation was found between granulysin level, disease activity, and severity.

Cathepsins are lysosomal proteases that play a role in many important biological processes, such as proteolytic processing of proenzymes, antigen presentation, inflammation, cell proliferation, differentiation, and apoptosis^28^. Cathepsin-L, which belongs to this group and plays a role in various phases of the innate and adaptive immune response, also has important functions in the regulation of epidermal homeostasis and the hair cycle^9,29,30^. Cathepsin L2, which has a very similar protein sequence to cathepsin-L, has been reported to be expressed at higher levels in keratinocytes from lightly pigmented skin than in darkly pigmented skin^31^.

CD 8+ T cells contribute significantly to cell death and tissue destruction in inflammatory skin diseases such as psoriasis and lichen planus^32^. In an animal experiment conducted by Yamada et al.^33^, to determine the effect of inhibiting cathepsin-L on T cell-mediated autoimmunity, they found that inhibition of cathepsin-L prevented the cytotoxic activity of CD8+ T cells in pancreatic islets in mice with autoimmune type 1 diabetes. They suggested that cathepsin-L may be a new target molecule that can be used in the treatment of type 1 diabetes.

In this study, although cathepsin-L levels were significantly higher in vitiligo patients than in the control group, the molecule concentration was not associated with disease activity and severity. However, it would be useful to investigate this issue in a prospective and large series of studies.

## CONCLUSION

The etiopathogenesis of vitiligo is still not clearly understood. Although granulysin and cathepsin-L are molecules that play a role in the etiopathogenesis of vitiligo, they may not be useful for the assessment of disease severity and activity. New molecules are needed to assess the activity, severity, and potential course of vitiligo disease.
